# Hand grip strength and loss of independence in Indigenous and non-Indigenous New Zealand octogenarians—the LiLACS NZ cohort study

**DOI:** 10.1093/gerona/glag066

**Published:** 2026-03-10

**Authors:** Simon A Moyes, Vanessa Selak, Lindsay D Plank, Joanna Hikaka, Ruth Teh, Ngaire Kerse

**Affiliations:** Department of General Practice and Primary Healthcare, School of Population Health, Faculty of Medical and Health Sciences, University of Auckland, Auckland, New Zealand; Department of Epidemiology and Biostatistics, School of Population Health, Faculty of Medical and Health Sciences, University of Auckland, Auckland, New Zealand; Department of Surgery, Faculty of Medical and Health Sciences, University of Auckland, Auckland, New Zealand; Waitemata District Health Board, Auckland, New Zealand; Te Kupenga Hauora Māori, Faculty of Medical and Health Sciences, University of Auckland, Auckland, New Zealand; Department of General Practice and Primary Healthcare, School of Population Health, Faculty of Medical and Health Sciences, University of Auckland, Auckland, New Zealand; Department of General Practice and Primary Healthcare, School of Population Health, Faculty of Medical and Health Sciences, University of Auckland, Auckland, New Zealand

**Keywords:** Trajectory groups, Epidemiology, Function, Sarcopenia

## Abstract

**Background:**

The number of people losing their independence is increasing as the population ages. Sarcopenia, low muscle strength and mass, and hand grip strength (HGS) are known to predict reduced independence in people in their seventies. This paper investigates these relationships in New Zealand octogenarians, including Indigenous Māori.

**Methods:**

This study used data from Life and Living in Advanced Age: A Cohort Study in New Zealand (LiLACS NZ), which recruited 421 Māori and 516 non-Māori in 2010-2011. The Nottingham Extended Activities of Daily Living (NEADL) scale measured independence. Participants were classified by ethnicity (Māori or non-Māori) and sex into high, medium or low independence trajectory groups based on their six annual NEADL scores using group-based trajectory modelling. The associations between HGS or probable sarcopenia (a binary measure of HGS) and independence trajectory group (high, medium, low) were tested separately by ethnicity and sex in multinomial logistic regression models.

**Results:**

Hand grip strength was inversely associated with low (versus medium) independence trajectory among Māori women, non-Māori women and non-Māori men, adjusting for age, comorbidities and cognition (adjusted odds ratio, aOR, 0.84 (95% CI 0.72, 0.97), 0.86 (0.77, 0.96) and 0.90 (0.81, 0.99), respectively). Similar associations were seen for probable sarcopenia and independence trajectory among those three groups, but the effects did not reach statistical significance.

**Conclusions:**

Hand grip strength could be a valuable screening tool to assess independence trajectory in octogenarians, but further study is needed to clarify the effects of indigeneity and sex in this population.

## Introduction

### Background/rationale

Preserving the ability to perform basic tasks is crucial to maintaining independence in older age. Sarcopenia, the loss of muscle mass and strength, can impact this ability and lead to reduced independence as gauged by instrumental activities of daily living (IADL) scales.^1–3^

The Second European Working Group on Sarcopenia in Older People (EWGSOP2) proposed using hand grip strength (HGS) to screen for probable sarcopenia.^4^ Low HGS is associated with low IADL and is predictive of its deterioration.^5–8^

Many studies dichotomise their IADL scores into ‘can do all’ or ‘cannot do at least one task’.^6^^,^^9^ One example of an IADL score is the Nottingham Extended Activities of Daily Living (NEADL) scale.^10^ The NEADL scale is scored out of 22 and can be utilized as a continuous measure. While many studies have looked at the link between either sarcopenia and independence or HGS and IADL, these studies have generally focused on people in their seventies rather than their eighties and have not included Indigenous people. The present study uniquely explores these relationships in an octogenarian New Zealand population, including the Indigenous Māori. Investigating how loss of independence can be predicted in the oldest old, particularly Indigenous people, may lead to the development and prioritization of preventative interventions in the future.

### Objectives

This paper examined the utility of HGS and probable sarcopenia in predicting independence in IADL, as measured by the NEADL scale, for community-dwelling Māori and non-Māori octogenarians in New Zealand over five years.

## Methods

All analyses were stratified by sex and ethnic grouping, which were self-defined in the initial interview.

### Study design

The data analyzed here are from LiLACS NZ, a birth cohort study conducted by researchers of the University of Auckland that followed non-Māori from their eighty-fifth year and Māori in their eighties.^11^

### Setting

The LiLACS NZ study recruited participants from the general population in the Bay of Plenty region of New Zealand between 2010 and 2011. There were six annual waves of interviews and physical examinations from 2010 to 2016. Both were usually conducted at the participant’s home or a dedicated assessment centre, occasionally at a primary healthcare clinic.

Written informed consent was requested from study participants to access routinely collected Ministry of Health (MoH) data. These data include admission and discharge dates and any diagnoses and procedures for all publicly funded hospital admissions in the country.

### Participants

All those living in the study area, either born in 1925 or self-identified as Māori and born 1920-1930, were eligible to participate in the LiLACS NZ study. The widened age range for Māori enlarged the population pool for the Indigenous participants to achieve a similar sample size to that of the non-Māori group.^11^ The recruitment birth year ranges were chosen from Statistics New Zealand’s life tables to have roughly 10% mortality per annum in 2010.^12^

Seven local primary healthcare and Māori providers undertook recruitment, interviews and physical assessments. The Electoral Roll was used as the basis for recruitment, and these organizations added those on their patient lists and advertised the study more generally to recruit participants. Each year after recruitment, a participant would be telephoned to arrange a follow-up interview and physical assessment unless they had previously declined to participate further or died.

Participants living in residential care were excluded from the analyses presented here as their domestic chores were carried out by facility staff regardless of the participants’ capabilities.

### Data sources/measurement

#### Outcome

Trajectory groups were created using all six potentially available NEADL assessments for each participant taken in the study. Mortality was high in both non-Māori and Māori, so only a minority of participants could be assessed in the sixth annual wave. This attrition resulted in the trajectory of each group being based on a smaller sample for each wave.

#### Predictors

Muscle strength was assessed using HGS as part of the physical assessment. The participant squeezed the handgrip of the Takei GRIP-D dynamometer (Takei Scientific Instruments Co., Ltd, Tokyo, Japan) whilst standing with their elbow at full extension and their arm at their side three times with each hand. The highest of the six readings was used as the HGS estimate. If the participant could not stand for the test, it was performed seated with the arm in the same position as if standing. Probable sarcopenia derived from HGS was also tested as a predictor. The cut-offs used are from the EWGSOP2 and are less than 27 kg for men and 16 kg for women.^4^

Participants’ age at their initial interview was controlled for in all models. Potentially fatal diseases and chronic conditions could be confounders, but were observed in numbers too small to be used singly. Instead, the Multimorbidity Measure (M3) score was used as a covariate. The M3 score is derived from hospitalization data, and its development team found that in New Zealand, the M3 outperformed the Charlson and Elixhauser indices in predicting mortality.^13^ The M3’s creators recommend using either a one or five-year history of hospitalizations; as LiLACS NZ had a relatively small sample size, the latter was selected.

Cognition could also impact independence^14^; in the LiLACS NZ study, the Modified Mini-Mental State Examination (3MS) was used. The 3MS is a hundred-point scale that assesses various aspects of cognition, including short and long-term memory, verbal fluency, and temporal and spatial orientation.^15^ For modelling, the 3MS was kept as a continuous measure.

### Bias

The LiLACS NZ team attempted to recruit as many people eligible for the study as practicable within the survey area. An examination of the differences between those recruited and those not has been published previously; non-Māori women and those in residential care were underrepresented in the total sample.^16^ However, non-Māori women were analyzed separately from the rest of the cohort, and those in residential care were excluded from the analyses presented here.

### Study size

The sample size for the LiLACS NZ study (approximately 500 Māori and 500 non-Māori) was chosen to detect a difference in mortality rate between different levels of nutrition risk or activities of daily living, with an expected overall mortality rate of 10% per annum.^11^ To increase recruitment, both the full and core questionnaires were offered to participants, and a further physical assessment was optional. In this paper, the participants were restricted to those who were community-living and completed both the full questionnaire (including the NEADL) and the physical assessment (including the HGS) at baseline.

### Statistical methods

Independence trajectory groupings were calculated by inverting the NEADL score, counting the number of activities not done to approximate a Poisson distribution. Māori and non-Māori, men and women participants were categorized into trajectory groups based on their inverted NEADL score over six waves using group-based trajectory modelling (GBTM). Differing numbers of trajectory groups were tested using GBTM, but more than three proved to have too few participants in the fourth group.

Baseline characteristics were described for each sex-ethnicity group, overall and by trajectory group (high, medium and low independence). Multinomial logistic regression models were then used to assess the association between baseline predictors and trajectory group membership. Independence could have been influenced by age, health and cognition, so measures of these confounders were included in modelling; first separately, then combined with HGS or probable sarcopenia. The medium independence group was used as the reference group, as it was the largest trajectory grouping.

All analyses were performed using SAS software, version 9.4 (TS1M5) for Windows (SAS Institute Inc., Cary, NC).

Group-based trajectory modelling (GBTM) utilized the PROC TRAJ package (http://www.contrib.andrew.cmu.edu/∼bjones/index.htm).

#### Missing data

Age, sex, and ethnicity were known for all participants. Some participants in the study did not have an initial physical assessment, so they did not have an initial HGS measurement. A few that had a physical assessment did not attempt a HGS measurement. The GBTM estimated trajectory grouping if any of the six NEADL scores were available; a few participants were excluded as they did not complete the NEADL at any stage.

## Results

### Participants

The study recruited 56% of eligible Māori and 59% of eligible non-Māori in the recruitment area (421/766 and 516/870, respectively).^17^ Of those recruited, 11% of Māori and 17% of non-Māori were excluded from these analyses as they were in residential care at some point during the study (48/421 and 90/516, respectively). A further 26% of Māori and 14% of non-Māori were excluded from these analyses as they completed the core questionnaire at baseline, which did not have the NEADL and were not expected to do the physical examination (110/421 and 71/516, respectively). Others did not complete the NEADL (3%, 11/421 of Māori; 1%, 4/516 of non-Māori) or the HGS (6%, 27/421 of Māori; 6%, 29/516 of non-Māori) despite completing the full questionnaire. When added together, these three prerequisites excluded 47% of Māori and 38% of non-Māori in the study, leaving a total of 547 participants (131 Māori women, 94 Māori men, 166 non-Māori women and 156 non-Māori men).

Participants dropped out of the study because of preference, relocating out of the study area, illness, or death. Death was the most common reason for withdrawal, followed by preference; the numbers of each have been published previously.^16^ Most of those included in analyses dropped out before wave 6, so all trajectories are less well defined for later time points (Table 1).

**Table 1:** Number of NEADL scores for each wave of the LiLACS NZ study by ethnicity and sex.

Wave	Māori women	Māori men	non-Māori women	non-Māori men
**Any**	131 (100%)	94 (100%)	166 (100%)	156 (100%)
**1**	127 (97%)	89 (95%)	163 (98%)	154 (99%)
**2**	98 (75%)	66 (70%)	147 (89%)	136 (87%)
**3**	72 (55%)	41 (44%)	121 (73%)	121 (78%)
**4**	57 (44%)	31 (33%)	102 (61%)	102 (65%)
**5**	39 (30%)	26 (28%)	87 (52%)	85 (54%)
**6**	35 (27%)	20 (21%)	73 (44%)	71 (46%)

Each wave represents an annual interview and assessment.

### Descriptive data

Women were 54%, and Māori 41%, of participants included in modelling (297/547 and 225/547, respectively). The mean age of Māori was 82 at baseline, younger than the non-Māori mean of 85 years. Men had a greater mean HGS than women (30.9 kg (SD 6.2 kg) compared to 19.3 kg (SD 4.7 kg), respectively; tested by generalized linear model, the age-adjusted *p*-value was <.0001). Mean HGS in Māori was slightly higher than in non-Māori for men and women (tested by generalized linear model; the age-adjusted *p*-value was =.67). Most study participants had a recent history of ill health; they had been hospitalized with some morbidity within five years of starting the study (55% had an M3 score greater than zero). Overall, 23% of those tested had probable sarcopenia (Table 2). The differences in sarcopenia rate were not statistically significant (tested by logistic regression; the smallest age-adjusted *p*-value was for Māori versus non-Māori women at 0.15).

**Table 2:** Baseline demographics for the LiLACS NZ cohorts.

	Māori women		Māori men		non-Māori women		non-Māori men	
	*N*	Mean (SD)	*N*	Mean (SD)	*N*	Mean (SD)	*N*	Mean (SD)
**Age (years)**	131	82.3 (2.6)	94	81.9 (2.5)	166	84.5 (0.5)	156	84.6 (0.5)
**Ethnicity^a^**								
Māori	131	131 (100%)	94	94 (100%)	166	0 (0%)	156	0 (0%)
European	131	66 (50.4%)	94	36 (38.3%)	166	165 (99.4%)	156	156 (100%)
Pacific	131	2 (1.5%)	94	0 (0%)	166	1 (0.6%)	156	0 (0%)
Asian	131	0 (0%)	94	2 (2.1%)	166	0 (0%)	156	0 (0%)
Other	131	0 (0%)	94	0 (0%)	166	0 (0%)	156	0 (0%)
**Education *n* (%) Secondary school qualification or higher**	129	44 (34.1%)	93	31 (33.3%)	164	76 (46.3%)	156	76 (48.7%)
**BMI (kg/m^2^)**	129	28.9 (5.5)	92	30.3 (5.2)	166	27.0 (4.1)	156	26.8 (3.7)
**NEADL (/22)**	127	18.0 (4.0)	89	17.3 (3.8)	163	18.7 (2.4)	154	18.4 (2.6)
**NEADL Median [IQR]**	127	19 [17, 21]	89	18 [15, 20]	163	19 [17, 21]	154	19 [17, 20]
**HGS (kg)**	131	20.0 (5.1)	94	31.0 (6.9)	166	18.7 (4.3)	156	30.8 (5.7)
**HGS Median [IQR]**	131	20 [17, 23]	94	30 [27, 34]	166	19 [16, 22]	156	31 [27, 35]
**Probable sarcopenia**	131	22 (16.8%)	94	21 (22.3%)	166	42 (25.3%)	156	39 (25.0%)
**No sarcopenia**	131	109 (83.2%)	94	73 (77.7%)	166	124 (74.7%)	156	117 (75.0%)
**SPPB (/12)**	123	8.2 (2.7)	86	8.3 (2.7)	147	7.9 (2.6)	151	8.8 (2.5)
**3MS (/100)**	128	91.0 (7.5)	89	87.2 (11.9)	163	93.3 (6.4)	151	92.4 (8.6)
**3MS <70/100 *n* (%)**	128	1 (0.8%)	89	6 (6.7%)	163	1 (0.6%)	151	3 (2.0%)
**Dementia^b^**	128	11 (8.6%)	89	14 (15.7%)	163	12 (7.4%)	151	12 (8.0%)
**M3 morbidity Score**	123	0.3 (0.5)	88	0.5 (0.8)	163	0.3 (0.6)	151	0.3 (0.6)
**Without morbidity *n* (%)^c^**	123	74 (60.2%)	88	38 (43.2%)	163	88 (54.0%)	156	93 (59.6%)
**PASE Score**	129	108.0 (72.6)	89	132.3 (93.2)	163	90.5 (48.2)	156	125.3 (72.5)
**Number of medications**	128	5.0 (3.2)	89	5.1 (3.3)	163	5.3 (3.2)	154	5.0 (3.3)
**Not on any meds *n* (%)**	128	12 (9.4%)	89	7 (7.9%)	163	9 (5.5%)	154	8 (5.2%)
**Smoking *n* (%)**								
Never	129	68 (52.7%)	94	26 (27.7%)	166	116 (69.9%)	156	59 (37.8%)
Current	129	16 (12.4%)	94	11 (11.7%)	166	3 (1.8%)	156	8 (5.1%)
Past	129	45 (34.9%)	94	57 (60.6%)	166	47 (28.3%)	156	89 (57.1%)

a Self-identified, more than one could be selected.

b 3MS, the Modified Mini-Mental State Examination, score below 80 for Māori, and 84 for non-Māori. The cut-offs were determined in a sub-study.^41^

c M3, Multimorbidity Measure, score of zero.

Abbreviations: *N*, total number of LiLACS NZ participants.

Age, age at time of first assessment (years).

BMI, body mass index (kg/m^2^).

NEADL, Nottingham Extended Activities of Daily Living, a 22-point scale with a score of 22 being the highest performance and 0 being unable to perform any task independently.

IQR, interquartile range.

HGS, hand grip strength (kg of force).

SPPB, Short Physical Performance Battery, a twelve-point scale with a score of 12 being the highest performance and 0 being unable to complete any test in the battery.

3MS, the Modified Mini-Mental State Examination a hundred-point scale of cognition with a score of 100 being the highest cognition and 0 being unable to answer any question correctly.

M3, Multimorbidity Measure scale with a score of 0 being no comorbidities. Applied to the previous five years hospitalizations.

PASE, the Physical Activity Scale for the Elderly with a higher score indicating greater activity.

### Trajectory groups

**Figure 1:**
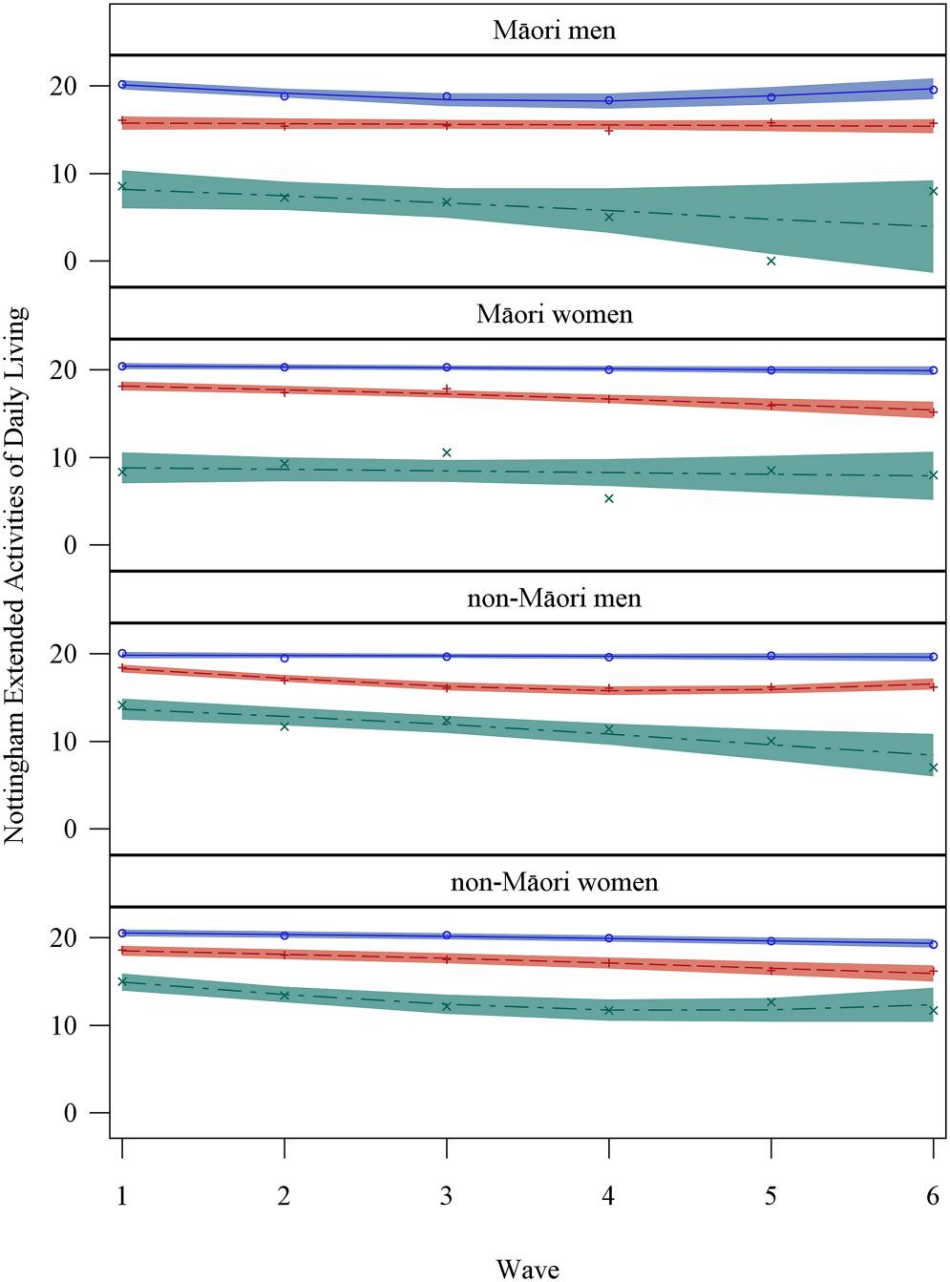
Independence trajectory grouping of the LiLACS NZ cohorts by sex and ethnicity over time. Note. Lines represent the modelled trajectory of each group, with a 95% confidence interval band about them. Observed mean scores for each trajectory group at each annual interview are indicated with circles. The high independence group is blue, the medium red and the low independence group green.

Although modelled separately, Māori and non-Māori men and women were divided into similar trajectory groups (Figure 1). The three trajectory groups for each ethnicity-sex combination were categorized as high, medium, or low independence. In all four ethnicity-sex combinations, the medium independence group was the largest (47-57% of the sample), with the high independence group being intermediate (29-40%) and the low independence group being the smallest (12-23%, Table 2).

For all four sex-ethnicity combinations, the high independence group started with almost total independence, having median NEADL scores of 20/22 or better. The medians for the medium independence scores were between 16 and 19, indicating some reliance on others for difficult tasks. The medians for the low independence scores were lower still, indicating much more reliance on others.

Generally, the participants’ independence, as gauged by the NEADL score, was slightly lower than their baseline after five years. The Māori women in the medium independence group had a statistically significant decline in independence over time, but high and low independence groups remained little changed over time. Function for Māori men in the medium independence group was stable, but there was a considerable deterioration over time for the low independence group. Māori men in the high independence group had an initial significant linear decline in independence over time but a significant quadratic element to their trajectory, indicating a recovery of independence after a few years. Non-Māori women in all three groups significantly declined in independence over time. However, their low independence group had a significant quadratic element to their trajectory, indicating a recovery of independence after a few years. The independence of non-Māori men in the medium and low groups significantly declined over time, with the medium group recovering independence after a few years. Function for non-Māori men in the high independent group was stable.

### Predictors

The three trajectory groups differed significantly in HGS and comorbidities (M3 score) for each ethnicity-sex group (Table 3). A similar pattern was seen for Māori men, although statistical significance was not reached. Cognition (3MS score) was also significantly different between independence groups for Māori men and women, with the higher independence groups having greater cognition on average. Probable sarcopenia was inversely related to the level of independence, but this effect only reached statistical significance for non-Māori men.

**Table 3:** Characteristics of the independence trajectory groups of the LiLACS NZ cohorts at baseline.

	High independence	Medium independence	Low independence	*p*-Value
***Māori women***				
***N***	52 (40%)	62 (47%)	17 (13%)	
**NEADL Median {range}**	21 {18-22}	18 {14-22}	7 {0-19}	
**HGS (kg)**	20.5 (5.2)	20.7 (4.6)	15.6 (4.6)	**0.002**
**Age (yrs.)**	81.7 (2.3)	82.7 (2.5)	83.4 (3.2)	**0.017**
**M3 score**	0.2 (0.5)	0.3 (0.4)	0.9 (1.0)	**<0.001**
**3MS score**	93.0 (5.6)	88.5 (9.8)	73.8 (30.0)	**<0.001**
**Probable sarcopenia**	7 (13%)	10 (16%)	5 (29%)	0.322
***Māori men***				
***N***	40 (43%)	47 (50%)	7 (7%)	
**NEADL Median {range}**	20 {19-22}	16 {12-20}	9 {0-16}	
**HGS (kg)**	33.0 (7.3)	29.8 (6.6)	27.9 (2.9)	0.058
**Age (yrs.)**	82.1 (2.7)	82.1 (2.5)	82.5 (2.9)	0.807
**M3 score**	0.4 (0.7)	0.6 (0.7)	1.0 (1.2)	0.089
**3MS score**	90.4 (6.2)	85.7 (12.6)	73.2 (18.6)	**0.007**
**Probable sarcopenia**	8 (20%)	11 (23%)	2 (29%)	0.856
***non-Māori women***				
***N***	55 (33%)	81 (49%)	30 (18%)	
**NEADL Median {range}**	21 {19-22}	19 {15-22}	15 {6-21}	
**HGS (kg)**	19.2 (3.8)	19.2 (4.4)	16.8 (4.3)	**0.026**
**Age (yrs.)**	84.6 (0.5)	84.5 (0.5)	84.7 (0.6)	0.336
**M3 score**	0.2 (0.4)	0.4 (0.7)	0.6 (0.7)	**0.016**
**3MS score**	93.2 (8.0)	93.6 (5.4)	92.4 (6.6)	0.624
**Probable sarcopenia**	11 (20%)	20 (25%)	11 (37%)	0.244
***non-Māori men***				
***N***	49 (31%)	86 (55%)	21 (14%)	
**NEADL Median {range}**	20.5 {17-22}	18 {14-22}	13.5 {10-19}	
**HGS (kg)**	31.5 (4.9)	31.5 (5.7)	26.5 (6.0)	**0.002**
**Age (yrs.)**	84.5 (0.5)	84.6 (0.5)	84.7 (0.5)	0.435
**M3 score**	0.1 (0.3)	0.3 (0.5)	0.6 (0.9)	**0.007**
**3MS score**	93.6 (5.1)	92.3 (9.7)	88.8 (9.0)	0.168
**Probable sarcopenia**	8 (16%)	21 (24%)	10 (48%)	**0.028**

Abbreviations: HGS grip strength, M3 comorbidity score, 3MS cognition score. All values presented as number (percent) or mean (standard deviation) unless otherwise specified. Significant differences highlighted in bold.

### Combined models

The statistical significance of HGS in predicting the independence trajectory group was reduced when combined with other significant predictors in a single model (Table 4). For Māori women, an increase in HGS lowered the odds of being in the low independence trajectory group (adjusted OR with CI of 0.84 [0.72, 0.97]) and an increase in cognition increased the odds of being in the high independence trajectory group (1.07 [1.01, 1.14]) compared to the medium independence trajectory group. For Māori men, cognition remained the sole significant predictor; an increase in cognition increased the odds of being in the high independence trajectory group (1.08 [1.02, 1.16]) compared to the medium independence trajectory group. For non-Māori women, an increase in HGS lowered the odds of being in the low independence trajectory group (0.86 [0.77, 0.96]) compared to the medium independence trajectory group. For non-Māori men, an increase in HGS lowered the odds of being in the low independence trajectory group (0.90 [0.81, 0.99]) compared to the medium independence trajectory group.

**Table 4:** Odds ratios of independence trajectory grouping by sex and ethnicity in combined models of the LiLACS NZ cohorts.

Predictive variable	Trajectory grouping outcome (ref Med.)	Māori women	Māori men	non-Māori women	non-Māori men
		***Model 1***	***Model 2***	***Model 3***	***Model 4***
**HGS**	High	0.99 (0.91, 1.07)	1.05 (0.98, 1.13)	1.00 (0.91, 1.08)	1.00 (0.93, 1.07)
	Low	0.84 (0.72, 0.97)*	0.97 (0.83, 1.14)	0.86 (0.77, 0.96)**	0.90 (0.81, 0.99)*
**Age**	High	0.88 (0.75, 1.03)	1.09 (0.90, 1.33)	1.21 (0.62, 2.33)	1.11 (0.55, 2.22)
	Low	1.00 (0.80, 1.26)	1.02 (0.68, 1.55)	1.21 (0.52, 2.82)	1.33 (0.47, 3.79)
**M3**	High	0.98 (0.37, 2.60)	0.82 (0.39, 1.74)	0.48 (0.18, 1.27)	0.45 (0.16, 1.27)
	Low	2.24 (0.84, 5.97)	0.70 (0.11, 4.33)	1.60 (0.85, 2.99)	1.39 (0.59, 3.29)
**3MS**	High	1.07 (1.01, 1.14)*	1.08 (1.02, 1.16)*	0.99 (0.94, 1.04)	1.02 (0.97, 1.08)
	Low	1.01 (0.93, 1.09)	0.96 (0.90, 1.03)	1.01 (0.94, 1.09)	0.97 (0.93, 1.01)
		***Model 5***	***Model 6***	***Model 7***	***Model 8***
**Sarcopenia (probable vs none)**	High	0.64 (0.20, 2.02)	0.86 (0.28, 2.68)	0.88 (0.38, 2.07)	0.62 (0.23, 1.66)
	Low	1.27 (0.26, 6.18)	0.64 (0.05, 7.53)	1.89 (0.75, 4.73)	1.41 (0.45, 4.42)
**Age**	High	0.88 (0.75, 1.03)	1.08 (0.89, 1.31)	1.21 (0.62, 2.34)	1.15 (0.57, 2.29)
	Low	1.04 (0.83, 1.30)	1.04 (0.69, 1.56)	1.23 (0.54, 2.80)	1.53 (0.55, 4.22)
**M3**	High	1.04 (0.40, 2.73)	0.78 (0.38, 1.62)	0.48 (0.18, 1.27)	0.51 (0.18, 1.42)
	Low	2.76 (1.05, 7.24)*	0.70 (0.10, 4.89)	1.52 (0.82, 2.82)	1.70 (0.78, 3.69)
**3MS**	High	1.07 (1.01, 1.14)*	1.09 (1.02, 1.17)*	0.99 (0.94, 1.04)	1.02 (0.96, 1.07)
	Low	1.01 (0.93, 1.09)	0.96 (0.89, 1.02)	1.00 (0.93, 1.08)	0.97 (0.92, 1.01)

Abbreviations: Med Medium independence group, M3 comorbidity score, 3MS cognition score, HGS hand grip strength. All models include age, M3 and 3MS as explanatory variables. Models 1-4 also include HGS, and models 5-8 also include probable sarcopenia.

*p*-Values: *p < .05; **p < .01; ***p < .001, otherwise not significant. Significant differences highlighted in bold.

Probable sarcopenia was not a statistically significant predictor in combined models. For Māori women, cognition and comorbidities remained significant; for Māori men, cognition was the sole significant predictor. For non-Māori women and men, no predictor remained significant.

## Discussion

### Key results

Nearly all the trajectory groups declined for the first few years, though some groups’ scores recovered. Those with high independence initially maintained it. Māori and non-Māori men in the lowest independence group at baseline had the greatest loss of independence.

Grip strength, comorbidities and cognition predicted independence trajectory over the next five years. In the combined model, both comorbidities and cognition mediated the relationship between independence and HGS. These factors could be the drivers for both sarcopenia and HGS. Dementia makes tasks more mentally challenging and reduces HGS through poorer neuromuscular signalling, increasing the need for assistance.^18^ Physical illnesses can weaken muscle, impacting both HGS and the physical ability to perform tasks independently.^9^^,^^19^^,^^20^

Probable sarcopenia was more common in the lower independence group, but was only a statistically significant predictor of trajectory group for non-Māori men. However, when comorbidities were adjusted for, sarcopenia was no longer a significant predictor for non-Māori men. Grip strength as a continuous measure was a better predictor than probable sarcopenia.

### Limitations

#### Data source

Participants in the study with missing data had a significantly higher mortality rate than others. This difference indicates that a disproportionate number had opted out of the full interview, part of the interview, or the physical assessment because of poor health.

The exclusion of these participants could have weakened the apparent relationship between poor health indicators, like HGS, and independence. In particular, those missing as because they were unable to perform the HGS test, unable to squeeze with either hand, or would need help with many tasks.

#### Outcome

The NEADL’s creators acknowledged some flaws in their design.^10^ The component questions are self-reported, rather than assessed. Although the questions are phrased as ‘do you’ rather than ‘can you’, respondents may answer affirmatively if they could do something rather than if they had carried out the activity. Conversely, many activities are household chores that some in perfect health may not do themselves. A later study noted that reading and writing depend on good vision and are more popular with those with higher education.^21^ They also noted that 81% of their participants did not wash dishes, possibly because many of their sample lived with younger family members who did many household tasks for them. Their sample was from Hong Kong and Taiwan, and the researchers noted that the NEADL valuing individual independence may reflect Western culture, which could be relevant in our study, which has different ethnic groups.

Excluding those placed into residential care removed a subset with very low independence; results do not apply to this population. However, entry into residential care would not follow a continuous trajectory; it would be a sudden change in circumstance.

#### Predictor

##### Hand grip strength

There is no universally agreed-upon method of measuring HGS; slightly higher HGS readings are observed with the test subjects standing with their arm at their side than when they were tested sitting down with their elbow at 90°.^22^ Readings can vary between equipment from different manufacturers.^23^ The EWGSOP2 cut-offs were from a paper that pooled data from twelve studies; most used the JAMAR dynamometer while seated, only the Newcastle 85+ used the same Takei device standing as used in LiLACS NZ.^24^ The authors checked but did not find that adjusting for the differing methods and devices was necessary when pooling the data in their paper. The LiLACS NZ study used the same dynamometer and methods with the same age group as Newcastle 85+, so the results should have a similar level of compatibility with other studies. The greater mean HGS observed in the LiLACS NZ study compared to the Newcastle 85+ is consistent with studies in Germany and the Netherlands, so it is plausibly due to population differences rather than measurement error.^25^^,^^26^ (The Newcastle 85+ study published a paper predicting disability trajectory group, but did not examine HGS as one of the predictors of group membership.^27^)

##### Probable sarcopenia

The EWGSOP2 team acknowledged that their selection of cut-offs for HGS and other measures was arbitrary and a topic of further research. The EWGSOP2 procedure for defining sarcopenia identifies probable sarcopenia using muscle strength. Then, it confirms the diagnosis by determining poor muscle mass through a body composition scan or bioelectrical impedance analysis.^4^ There are reasons for low strength other than sarcopenia, such as degraded neuromuscular signals.^18^ However, in the LiLACS NZ study, few participants had dementia, and excluding those with a 3MS below 70 (Table 2) had a negligible effect on the models.

### Interpretation

Previous studies have noted that low HGS corresponds to limited IADL and is predictive of its deterioration.^5–7^ Sarcopenia has also been observed to predict reduced IADL.^1^^,^^2^ For Māori and non-Māori, men and women, HGS proved predictive of independence trajectory over five years. The participants with the strongest HGS at baseline, for their sex and ethnicity, generally maintained independence over the follow-up period. Probable sarcopenia, as a dichotomous variable, was not as predictive. For both ethnic groups and sexes, chronic illness was more predictive of low independence in the future than HGS. For Māori men and women, high cognition was more strongly predictive of maintaining independence. The collinearity of HGS, comorbidities and cognition meant that they all lost significance in the combined models. Cognition was the best stand-alone predictor for Māori, and the M3 comorbidity score was the best for non-Māori.

However, measuring a patient’s HGS would be quicker than testing their cognition or medical history in a clinical setting. A validated HGS cut-off would assist decision-making. The proportion of those with probable sarcopenia was higher in the low independence group than the high independence group for both sexes and ethnicities. A larger sample might have established probable sarcopenia as predictive of independence, so there is a need for further research.

### Generalizability

The results here indicate that for both Māori and non-Māori in their eighties, HGS can be used to assess their current independence and the potential decline of that independence over the next five years. Grip strength can be used to gauge the independence trajectory of community-dwelling octogenarians. Those with low HGS could be identified and treated to help maintain or even improve their independence. Effective treatment of sarcopenia and low strength in older populations is a topic of much ongoing research. Various dietary supplements, exercise regimen and medications have been proposed.^28–40^ The benefits of screening with HGS could be investigated further as a potential low-cost, simple-to-administer screening tool. Catching sarcopenia early and starting treatment before problems manifest could help reduce healthcare inequities between Māori and non-Māori.

## Data Availability

The data supporting this study’s findings are not openly available due to the permissions granted to the study investigators by the study participants. They are available from the corresponding author upon reasonable request. Data are in secure storage at the University of Auckland.
